# Role of artificial intelligence in risk prediction, prognostication, and therapy response assessment in colorectal cancer: current state and future directions

**DOI:** 10.3389/fonc.2023.1065402

**Published:** 2023-01-25

**Authors:** Arian Mansur, Zain Saleem, Tarig Elhakim, Dania Daye

**Affiliations:** ^1^ Harvard Medical School, Boston, MA, United States; ^2^ University of Ottawa, Ottawa, ON, Canada; ^3^ Department of Radiology, Massachusetts General Hospital, Boston, MA, United States

**Keywords:** artificial intelligence, radiomics, deep learning, machine learning, colorectal cancer

## Abstract

Artificial Intelligence (AI) is a branch of computer science that utilizes optimization, probabilistic and statistical approaches to analyze and make predictions based on a vast amount of data. In recent years, AI has revolutionized the field of oncology and spearheaded novel approaches in the management of various cancers, including colorectal cancer (CRC). Notably, the applications of AI to diagnose, prognosticate, and predict response to therapy in CRC, is gaining traction and proving to be promising. There have also been several advancements in AI technologies to help predict metastases in CRC and in Computer-Aided Detection (CAD) Systems to improve miss rates for colorectal neoplasia. This article provides a comprehensive review of the role of AI in predicting risk, prognosis, and response to therapies among patients with CRC.

## Introduction

Colorectal Cancer (CRC) is the third most common cancer worldwide in men and the second most common cancer in women with over 1.93 million new diagnoses in 2020 (1). CRC is also the second leading cause of cancer death worldwide with an estimated 935,173 deaths in 2020 (1). Roughly 50% of patients with CRC will develop liver metastasis throughout the course of their disease (2). Projections for 2040 estimate an increase in the global incidence of CRC with an estimated 3.2 million cases (3). In this era of personalized medicine, increased efforts are needed for more effective diagnosis, risk prediction, prognostication, and prediction of treatment response.

Artificial intelligence (AI) is a novel branch of computer science that involves the ability of computer systems to emulate the human behavior that requires intelligence, like thinking and decision-making. AI was born in 1956 through the work of the Darmouth AI conference and has since revolutionized various industries, including medicine (4). Through the development of AI, came various systems such as Machine learning (ML), which is a subset of AI that involves a range of statistical, probabilistic, and optimization models that allows a computer system to learn and adapt from previous iterations to analyze and make inferences from patterns in noise (5, 6). Deep learning (DL) is a subset of ML that employs neural networks with multiple layers of processing to analyze and learn in a similar fashion to humans (7).

One of the exciting applications of AI has been in the field of radiology, which involves the use of imaging to diagnose and treat illnesses, and especially radiomics. Radiomics is a process that refers to the extraction of mineable data from medical images (8). Its importance is based on the fact that a vast number of extracted features, like entropy patterns, skewness, and kurtosis are mostly hidden to the human eye and can inform clinical decision making, quantify tumor phenotype, predict response to treatment, and determine prognosis. AI is not only able to effectively analyze the features generated by radiomics but also analyze the images to create its own radiomic features to forego predefined features, which is conventionally known as deep radiomics (9, 10). Given that radiology intersects with several fields of medicine, the advancements of AI in radiology has influenced several types of medical fields like oncology, including CRC.

**Figure 1:**
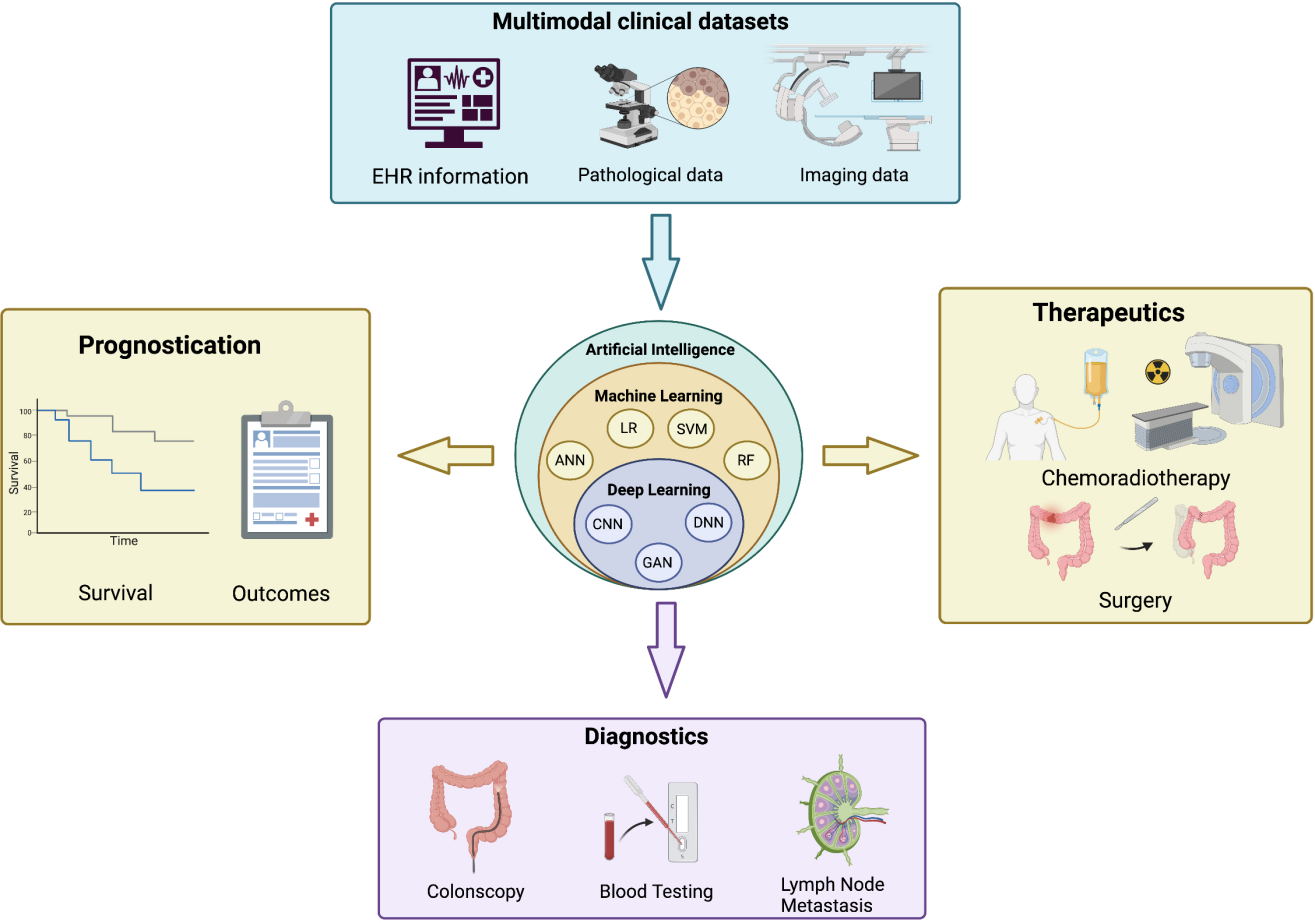
An overview of the applications of artificial intelligence in colorectal cancers. (Top) Input features from multimodal clinical datasets are integrated into artificial intelligence algorithms (center) that provide key information on the prognostication (left), diagnostics (bottom) and therapeutics (right) of colorectal cancer. EHR, electronic health record; ANN, artificial neural network; LR, logistic regression; SVM, support vector machine; RF, random forest; CNN, convolutional neural network; GAN, generative adversarial network; DNN, deep neural network. Created with BioRender.com.

The management of patients with CRC is multifaceted and involves various dimensions of care, such as screening, diagnosis, treatment, and follow-up. Because of these multiple components of care and the vast capabilities of AI, these technologies have an incredibly important role in the management of CRC, anywhere from polyp detection to the prediction of response to chemoradiotherapy. Various applications are currently being developed and validated to improve the detection and workup of CRC treatment. **Figure 1** shows an overview of the various features considered in these AI models and some of the applications specific to CRC. **Table 1** prevents a summary of the major studies of AI in CRC. In this article, we explore the role of artificial intelligence-based technique in risk prediction, prognostication and therapy response assessment in colorectal metastasis.

**Table 1:** Summary of major studies of AI in CRC.

Section	Ref	Year(s)	Type of study	*N*	Modalities	Major findings
Colonoscopy	Hassan et al. (11)	2019-2020	Systematic review and meta-analysis	5 RCTs (4354 patients)	CADe	Pooled ADR and APC were significantly higher in the CADe group than in the control.
Colonoscopy	Huang et al. (12)	2019-2021	Systematic review and meta-analysis	10 RCTs (6629 patients)	CADe, ENDOANGEL (13), AQCS (14)	ADR and PDR were significantly higher in AI-guided groups over routine colonoscopy.
Histopathology	Lui et al. (15)	2010-2019	Systematic review and meta-analysis	18 studies (7680 images)	Region growing algorithm, Computer-based algorithm, SVM, Retrieval-based software classification, WavSTAT4, CNN model, Gaussian mixture model, Color intensity analysis software, Endobrain	Histology prediction of diminutive polyps had a pooled AUC of 0.98. There was no significant difference between AI and expert endoscopists in accuracy on polyp histology prediction, but AI was significantly better than nonexpert endoscopists.
Histopathology	Thakur et al. (16)	2015-2020	Systematic review	30 studies with 40 models	AlexNet, ResNet, CNN, VGG, RNN, DCRN, R2U-Net, FCN, LSM, LSTM	Studies analyzed gland segmentation, tumor classification, tumor microenvironment characterization, and prognosis prediction with promising results but datasets had relatively limited scale for clinical application.
Histopathology	Davri et al. (17)	2016-2022	Systematic Review	82 studies	CNNs and GANs with various customizations	17 studies examined diagnosis, 17 on classifying tumor tissues, 19 on investigating tumor microenvironment, 14 on prognosis, metastasis, and survival from histological features, and 10 on microsatellite instability status.
Blood Testing	Ginghina et al. (18)	2022	Review	323 references	Various deep learning and machine learning algorithms	Various applications of AI and liquid biopsy in CRC are discussed.
Imaging	Nazarian et al. (19)	2003-2020	Systematic review and meta-analysis	48 studies	SVM, shape-UCM, CAD, EndoBRAIN, SfM, CNN, DNN, segmentation algorithm, DeepLab framework, ResNet, CWC, and others	Majority of studies that focused on polyp characterization had sensitivities, specificities, and accuracies above 82%. AI had significantly greater pooled ADR and PDR compared to colonoscopy.
Metastasis Detection	Bedrikovetski et al. (20)	2016-2020	Systematic review and meta-analysis	17 studies (12 in meta-analysis)	Radiomics and Deep Learning methodologies	Diagnostic accuracy for lymph node metastasis detection on pre-operative staging imaging was analyzed with per-patient AUC of 0.81 and 0.92 in radiomics and deep learning models for rectal cancer, which was better than the radiologists (AUC of 0.69). Both models were also better than the radiologist in colorectal cance.r
Predicting Mutation Type/Microsatellite Instability	Park et al. (21)	2018-2021	Systematic review	13 studies	Inception-V3, ResNet, ShuffleNet, MSInet, InceptionResNetV1	Prediction of microsatellite instability based on tumor histomorphology was evaluated. In CRC, AI-based models showed performance with the highest standard of 0.972, but most studies had on or more high-risk factors regarding bias.
Prediction of Survival	Staal et al. (22)	2007-2020	Systematic review	76 studies	Radiomics	19 studies focused on survival prediction with heterogenous results. Homogenous tumors were associated with better survival.
Personalizing Treatment	Aikemu et al. (23)	2017-2018	Observational study	250 patients	Watson for Oncology	The clinical recommendations from Watson for Oncology were highly matched with the multidisciplinary team (concordances for colon cancer, rectal cancer, or overall were 91%).
Primary Colorectal Cancer	Wesdorp et al. (24)	2015-2019 (for primary CRC)	Systematic review	27 studies for primary CRC	Radiomics (machine learning classifiers like RF, SVM, ANN, and DNN were often utilized)	Methodology greatly varied. In 16 of the 17 studies that constructed radiomics-based prediction models for response to treatment in patients with primary CRC, there was good discriminative power with AUCs 0.72-0.98.
Metastatic Colorectal Cancer	Wesdorp et al. (24)	2016-2019 (for metastatic CRC)	Systematic review	6 studies for metastatic CRC	Radiomics	Three of five studies that analyzed individual radiomic features showed good discriminative power with AUCs 0.74-0.81, while two showed no association.
Metastatic Colorectal Cancer	Russo et al. (25)	2007-2022	Systematic review and meta-analysis	26 studies (10 studies in meta-analysis)	Radiomics using various machine learning models	AI-based methods to predict response to chemotherapy alone or with targeted therapy in patients with metastatic CRC was analyzed. Overall weighted means of the AUCs were 0.90 and 0.83 in the training and validation sets.
CRC Surgery	Anteby et al. (26)	2016-2020	Systematic review and meta-analysis	32 studies (4 studies in meta-analysis). 3004 videos	BN-GoogLeNet, Mask Regional CNN, ResNet, Faster R-NN, Attention-guided Network, AlexNet, YOLOv3, 3D CNN, Inception-ResNet, Spatio-tempora-Net, Caffle framework	Various applications of AI in CRC surgery—instrument recognition and detection, phase recognition, anatomy recognition and detection, action recognition, surgery time prediction, and gauze recognition. Pooled sensitivity of 0.93 and specificity of 0.96 in meta-analysis.

RCT, Randomized controlled trial; CADe, Computer-aided polyp detection; ADR, adenoma detection rate; APC, adenoma per colonoscopy; AQCS, Automatic quality control system; PDR, polyp detection rate; CNN, convolutional neural network; SVM, support vector machine; RNN, recurrent neural networks; DCRN, densely connected recurrent convolutional network; R2U-Net, recurrent residual U-Net; FCN, fully convolutional networks; LSM, locality-sensitive method; LSTM, long short-term memory; GAN, generative adversarial network, CAD, computer-aided diagnosis; SfM, structure from motion; DNN, deep neural network; CWC, color wavelet covariance; RF, random forest; ANN, artificial neural network.

## Role of AI in CRC diagnostics

### Colonoscopy

The prognosis of CRC appears to be substantially improved when detected in its early stages (18). Screening can reduce the mortality and incidence associated with CRC by facilitating early diagnosis and treatment (27). In fact, colonoscopy may lead to a 90% risk reduction for CRC by allowing practitioners to identify suspicious lesions in the walls of the intestine (28, 29). This is particularly important as most adenomatous polyps are asymptomatic (29). However, although colonoscopy is the gold standard for diagnosing neoplastic lesions, it is accompanied by limitations including adenoma miss rates (AMR) ranging from 6% to 28% (18, 30). In fact, more than half of CRC cases which occurred post-colonoscopy arise from lesions that were missed during prior colonoscopies (31).

The ability to detect lesions during colonoscopy can vary significantly based on several factors, including operator performance (29). In addition, polyps which are flat, depressed, or have a diameter smaller than 10 mm may be harder to detect as colonoscopy is dependent on clinical experience and relies on the practitioner’s ability to visually identify the lesion, which is a complex and error-prone task (18, 27, 32). Other factors such as the size of the tumor, its patterns of enhancement, and variability in clinical symptoms can further add to the complexity of making diagnostic decisions (28, 33).

AI algorithms have been shown to augment the accuracy of existing endoscopic screening procedures. For example, computer-aided detection (CADe) can be used during colonoscopy to increase adenoma detection rates by highlighting abnormal areas of an image (18, 32). One systematic review and meta-analysis consisting of five randomized clinical trials (RCTs) found that adenoma detection rates (36.6% vs. 25.2%, *P* < 0.01) and overall polyp detection rate (50.3% vs. 34.6%, *P* < 0.01) were significantly higher in the CADe group compared to the control group (11). In another meta-analysis of 10 RCT, adenoma detection rates (35.4% vs. 24.9%, *P* < 0.001) and polyp detection rates (48.6% vs 33.8%, *P* < 0.001) were significantly higher when compared with routine colonoscopy (12).

During colonoscopy, computer-aided diagnosis (CADx) algorithms can be used to characterize the region based on the probability of its malignancy (18, 32). Kambe et al. conducted a randomized control trial to investigate whether a CADe model could reduce AMR during colonoscopy (34). Compared to the standard colonoscopy group which had an AMR of 36.7%, the patients who received CADe-assisted colonoscopy had a significantly lower AMR of 13.8% (*P* < 0.01). Brown et al. investigated AMR, sessile-serrated lesion (SSL) miss-rate and adenomas per colonoscopy (APC) in patients who underwent high-definition white light colonoscopy (HDWL) versus colonoscopy assisted with CADe (35). This randomized control trial found significant lower AMR (20.1% vs. 31.3%, *P* = 0.025), lower SSL miss-rate (7.1% vs. 42.1%, *P* = 0.048), and higher first-pass APC (1.19 vs. 0.90, *P* = 0.03) in the CADe group compared to the HDWL group. Interestingly, another randomized control trial found that CADe-assisted colonoscopy significantly increased adenoma detection rate compared to the control group (53.3% vs. 44.5%, *P* < 0.01) (36). Other programs such as supervised learning (SL) algorithms can predict clinical outcomes after being trained using pre-labeled endoscopic images (18). In addition, various algorithms using convolutional neural networks have also been used for tumor identification, and the emergence of AI-based computed tomography (CT) colonography has further enabled the detection of flat neoplasms with greater accuracy (27).

Many polyps are missed during current practice, and they may present as precursors to CRC (37). Deep learning (DL) algorithms can enhance adenoma detection due to its ability to efficiently detect premalignant polyps (18). Such detection begins with adequate bowel preparation before colonoscopy, which is a pertinent step for the visualization of premalignant polyps. A recent study found that a novel AI based CNN model have a higher Boston bowel preparation scale and, therefore, higher quality of bowel preparation for detecting polyps compared to current routine practice (38). Polyp detection and localization systems that operate in real time are extremely useful in clinical practice and as of 2022 there has been 20 out of 43 studies with the ability to operate as such (39). Wang et al. were the first to perform a prospective research trial with polyp detection pipelines and had a frame-based evaluation (*F*_1_) of 0.91 (40). Recently a large database composed of 28,576 images were modeled into a DL pre-trained YOLOv3 (You Only Look Once) network for real time adenoma detection. The pipeline had an (*F*_1_ ) of 0.88 and a sensitivity reaching 90% for detecting and localizing polyps. Additionally Li K. et al. created a large CNN model for both polyp detection and classification, and had a sensitivity reaching 91% and 70%, respectively (41). Ozawa et al. used over 27,000 endoscopic images to design a CNN algorithm that can detect and classify polyps with a detection sensitivity of 0.92 and a classification sensitivity of 0.83 (42). Another CNN algorithm designed by Akbari et al. can perform polyp segmentation and produce an accurate probability map with a 99.3% sensitivity (43). A faster region-based CNN (Faster R-CNN) combined with a single shot multibox detector (SSD) was also used to detect polyps with a precision 0.8154 (44). Similarly, Godkhindi et al. used 825 CT images to develop a CNN algorithm for polyp detection (45). The resulting model could detect polyps with an accuracy of 0.87. In addition, RetinaNet can automatically localize polyps with a precision of 0.537 using a CNN-based algorithm (46). Moreover, as shown in the Medical Image Computer and Computer Assisted Intervention (MICCAI) 2015 Endoscopic Vision Challenge, the methods utilizing AI outperformed those that relied on manually extracted features (47) These AI-based algorithms have proven to be more advantageous than traditional radiomics methods that may be susceptible to errors as they rely on tumor segmentation and subsequent identification of clinically pertinent radiomic features.

That said, its apparent that incorporating AI into routine colonoscopy can decrease adenoma miss rates (AMR) through better detection, identification, and localization of the tumors, especially small ones <10mm, which has achieved a 50% reduction in a recent multicenter, multicounty study (31). This shows that AI-driven colonoscope approaches has important future implications on CRC screening moving toward better risk stratification, prognostication, and improvement of patient outcomes. However, further research is still needed in this area, especially to see if such improvements in adenoma recognition is not as same as a better mucosa exposure for adenoma detection.

### Histopathology

Histopathological characterization is often the last step in the diagnosis of CRC and is based on the texture, structure, and morphological features of the tissue (48). Precise tumor classification is vital for prognostication and is particularly important as even experienced pathologists may often disagree on the grading of tissue (49). AI applications may be useful in this step by preventing diagnostic inconsistency and improving inter- and intra-observer variability. One application of a hybrid deep learning (hDL) algorithm was used to distinguish low-grade CRC from high-grade CRC lesions with an accuracy of 99.1% (48). Moreover, a DL algorithm was used to automatically classify tissues without the need for segmentation (49). This model was able to differentiate between normal mucosa, hyperplastic polyps, adenoma, and adenocarcinoma with an accuracy of 80% (49). Another DL algorithm used ResNet to classify colorectal polyps on whole slide images (50). This program was shown to differentiate between hyperplastic polyps, sessile serrated polyps, traditional serrated adenoma, tubular adenoma, and villous adenoma with an accuracy of 93%. One meta-analysis found that AI could improve histology prediction, including those involving diminutive polyps, with a pooled specificity of 89.8% and sensitivity of 92.3% (15). In another systematic review focused on CRC pathology image analysis using artificial intelligence found that while applications were still in early stages, the results were still promising with respect to accurately diagnosing CRC (16). Furthermore, in another systematic review focused on the use of deep learning for the diagnosis of CRC *via* histopathological images found that various studies have promise in aiding the diagnosis, predicting relevant molecular features, identifying prognostic features with correlations to metastasis, and assessing tumor microenvironments (17).

CNN-based algorithms can also enable precise image classification; in fact, these algorithms have been shown to more accurately classify colorectal tumors when compared to endoscopists (51). Iizuka et al. used a CNN-based algorithm to automatically classify tissues as non-neoplastic, adenoma, or adenocarcinoma (52). Using annotated whole slide images, a CNN program was trained by a max-pooling method and a recurrent neural network (RNN). The resulting program could differentiate the tissues with an AUC of 0.96 for adenocarcinoma and 0.99 for adenoma.

Moreover, SL algorithms can be applied to support vector machines (SVMs) for the grading of unlabeled biopsies. One SVM algorithm was employed by Takemura et al. to discriminate neoplastic from nonneoplastic lesions with a high predictive power and detection accuracy of 97.8% (53). CADx algorithms can also be used to differentiate neoplastic from non-neoplastic polyps. Tamai et al. utilized a CADx algorithm in conjunction with narrow-band imaging (NBI) to accurately classify and differentiate between hyperplastic lesions, adenocarcinomas, and submucosal lesions (54). Similarly, Chen et al. used neural networks (NNET) to detect hyperplastic and neoplastic polyps that are smaller than 5 mm with 96.3% sensitivity (55).

In conclusion, several AI based algorithms has shown promise on accurate histopathologic diagnosis with some showing high performance on pathologic image analysis (56). These new techniques can ease the identification of cancer status in an accurate manner, which can eventually revolutionize the prediction performance in a way that may exceed the accuracy of pathologist analysis.

### Blood testing

CRC can also be detected using noninvasive procedures including blood testing. These procedures may be advantageous over endoscopic procedures such as colonoscopy which requires bowel preparation, carries a risk of bowel rupture, and is not indicated in patients with peritoneal irritation and anorectal stenosis (57). Pan et al., evaluated the predictive value of serum glycomic profiling in tandem with AI algorithms to identify advanced colorectal adenomas (57). In this study, the biomarkers of interest were multi-antennary N-glycans and core-fucosylated N-glycans, which were positively and negatively correlated with CRC stage, respectively. Subsequently, an algorithm which utilized random forests, logistic model trees (LMT), and SVM programs was employed to identify those patients who were at an advanced stage of CRC with an accuracy of 75%.

Moreover, Ivancic et al. investigated various serum protein biomarkers to identify those that were indicative of CRC (58). Using machine learning and SVM algorithms, it was shown that five biomarkers were strongly predictive of CRC, including superoxide dismutase 3, leucine-rich alpha-2-glycoprotein 1, inter-alpha- trypsin inhibitor heavy-chain family member 4, hemopexin, and epidermal growth factor receptor, with a specificity of 70% and a sensitivity of 89%. In one study, an AI learning algorithm (ColonFlag™) was used in patients with iron deficiency anemia who underwent a fecal immunochemical test. The study found that the AI learning algorithm may improve the prioritization of urgent referral as it was able to reduce the prioritization of patients from 592, who were referred based on their hemoglobin concentration, to 304 (59). A recent review summarizes various studies that utilize liquid biopsy and AI in CRC in order to detect signatures of colorectal malignancies and aid in stratification (18).

With such advancement in AI-based blood testing to diagnose CRC, it becomes clear that the future holds promise toward fewer procedural screening to accurately diagnose and identify high risk CRC, which can become an efficient alternative method to decrease procedural complications and improve patient flow.

### Imaging

Molecular imaging can be crucial in the practitioners’ ability to make effective decisions regarding the detection, diagnosis, and staging of colorectal tumors, in addition to predicting response to therapy (60). Existing molecular imaging techniques can be improved with the use of AI algorithms. For instance, feature-detection algorithms used in conjunction with magnifying chromoendoscopy has been shown to improve the discernment of neoplastic from non-neoplastic lesions (18). In addition, SVMs can be applied to endocytoscopy procedures to improve the discrimination of between benign lesions and neoplastic carcinomas (18). A systematic review and meta-analysis found that AI could improve the characterization of polyps after detection, with a sensitivity of 92% and accuracy of 87% (19).

AI algorithms can use confocal laser endomicroscopy (CLE) to augment the detection of polyps (61). CLE is a useful imaging technique that can identify subcellular features of the gastrointestinal mucosa due to its high magnifying capabilities (62). One AI model used the k-nearest neighbor algorithm to discriminate malignant lesions from benign lesions with 90% accuracy (63). In more recent developments, Stefanescu et al. used CADx algorithms in conjunction with CLE to more readily identify morphometric and malignant characteristics of the mucosa, which can aid in the diagnosis of CRC (61).

For patients that have a higher risk of complications associated with colonoscopy and those that cannot tolerate sedation, an alternative method of CRC screening is possible, including colon capsule endoscopy (CE) (18, 64). Colon CE is a minimally invasive procedure that allows practitioners to obtain images of the gastrointestinal lumen. This imaging procedure can also be augmented using AI algorithms. For instance, CNN algorithms have been applied to colon CE procedures which has allowed for the improved identification and localization of polyps with a high degree of sensitivity and specificity (28).

With further advancement in AI- based imaging modalities, we anticipate a paradigm shift toward improved image-guided detection of CRC, leading to fewer procedural interventions and complications. This can potentially increase patient convenience and medical cost, while keeping precision medicine intact.

### Metastatis detection

Lymph node metastasis (LNM) is the most common method for the spread of CRC (65). The precise assessment of LNM is vital for making clinical decisions as it can help establish the most effective treatment for CRC (66). However, LNM is cumbersome to predict before surgery, as the current standards for staging such as CT have an accuracy of 56.5% (67). Other techniques such as magnetic imaging resonance (MRI) can also be used to determine nodal staging; however, this also has a low diagnostic accuracy of 63% and a relatively low sensitivity (65, 67). To this end, current research shows that AI programs may be used to facilitate treatment decisions by improving the prediction of LNM. Liu et al. designed a multimodal multiple instance learning (MMIL) algorithm to predict lymph node metastasis (67). The MMIL was designed using a two-pronged approach: first, tumor-specific serum biomarkers (including CEA, CA125, CA19-9, and AFP) were inputted into a feed-forward network. Subsequently, this information was integrated with a feature extraction tool based on annotated whole slide images of the tumor microenvironment. The resulting MMIL model could predict lymph node metastasis with an area-under-the-curve (AUC) of 0.93, 0.88, 0.81, and 0.86 for stages T1, T2, T3, and T4, respectively. One systematic review and meta-analysis found that AI could improve pre-operative staging of lymph nodes and lead to a more accurate prediction of metastasis compared to traditional radiomics models (20).

Another algorithm applied five ML models to predict LNM, including SVM, random forests (RFs), NNET, logistic regression and extreme gradient boosting (66). The latter two models could predict LNM with an AUC of 0.87 and 0.90, respectively. Moreover, both of these algorithms were more effective in predicting LNM when compared to standard ^18^F-FDG PET/CT (^18^F-Fluorodeoxy Glucose Positron Emission/Computed Tomography) imaging. Ding et al. utilized a DL algorithm that could target specific features of 414 MRI images to predict metastatic lymph nodes (65). Subsequently, a faster R-CNN model was applied which resulted in LNM prediction with an AUC of 0.91.

In addition, AI algorithms can also be used to determine liver metastasis in CRC, which is a significant contributor to mortality in these patients. Kiritani et al. applied probe electrospray ionization-mass spectrometry in combination with ML to identify malignant spectrum patterns from tissue samples (68). The resulting algorithm was shown to predict liver metastasis with an accuracy of 99.5%.

Such progressive advancement in AI based metastatic detection has the potential to make the staging of CRC easily achievable with high accuracy, leading to a more efficient, personalized, and precise therapeutic approach. These advancements hold promise to the patient and the healthcare system as a whole due to a more efficient diagnostic processes while accurately individualizing patient care.

In conclusion, many AI-based applications holds promise toward the diagnoses of colorectal cancer through effective polyp detection, localization, identification, segmentation, and classification, which can eventually decrease adenoma miss rates and better predict clinical outcomes. The expansion of such approaches to accurate blood and histopathologic diagnoses, along with the use of various image-guided modalities to accurately determine the stage of CRC, has an overall ability to improve diagnostic variability and can revolutionize the current prognostication systems for a precise therapeutic approaches and better patient outcomes.

## Cancer genetics: prediction of mutation type and microsatellite instability

The carcinogenesis of CRC can be explained by several different molecular pathways, including the chromosomal instability pathway, microsatellite instability pathway, and the CpG island methylation pathway. In particular, microsatellite instability (MSI) is a key biomarker in CRC and is linked to deficient DNA mismatch repair (69). This indicator is very useful when making treatment decisions; for instance, it is used to screen for Lynch syndrome and patients with advanced CRC who present MSI are eligible to receive immune checkpoint blockade therapy (69, 70). MSI status is also useful after surgery when clinicians are selecting adjuvant chemotherapy. Existing tests for MSI status rely on methods that are resource-intensive and not readily available in most treatment facilities, including PCR analysis of microsatellite markers (69).

Several studies have shown that AI algorithms may be useful in detecting MSI. For instance, Echle et al. trained deep neural networks (DNNs) to detect MSI using hematoxylin-eosin (HE) tissue slides (69). The model could successfully identify MSI status with an AUC greater than 0.85 in eight out of the nine cohorts tested. Cao et al. used an integrated AI model—Ensemble Patch Likelihood Aggregation model (EPLA)—to predict MSI status based on whole slide images from the Cancer Genome Atlas (TCGA-COAD) cohort and the Asian CRC cohort (Asian-CRC) (70). An MSI-sensor algorithm assigned MSI status using paired genome sequencing based on MSI-sensor scores greater than or equal to 10. Furthermore, distinct microsatellite loci were analyzed using capillary electrophoresis to classify the cohort into MSI-high and MSI-low groups. A CNN-based algorithm—Resnet-18—was used to generate patch-level prediction of MSI, and the resulting data was inputted into the Patch Likelihood Histogram (PALHI) pipeline and the Bag of Words (BOW) pipeline. The combination of both pipelines using ensemble learning allowed for the prediction of MSI with an AUC of 0.89 in the test cohort.

In addition, AI algorithms can also be used in other genetic applications; for instance, a CNN model was used to predict tumor mutational burden-high (TMB-H) with an AUC of 0.93 (71). Moreover, AI has been shown to detect the presence of the KRAS proto-oncogene which may be implicated in the pathogenesis of CRC (72). In fact, 65% of carcinomas in the colon have been linked to mutations in the RAS family of genes, which includes the KRAS proto-oncogene (73). Identifying whether a patient has a mutated KRAS gene may be crucial as some individuals with this mutation may not be responsive to existing therapies, including anti-EGFR agents. To this end, one study conducted by Gonzalez-Castro et al. utilized several ML algorithms including SVM, Grade Boosting Machines, NNET, and RFs to identify if the KRAS gene is mutated (72). After extracting textural characteristics from CT images, the algorithms were able to classify the images as KRAS positive or negative. It was found that NNET in conjunction with Haralick texture analysis was most efficient, with an accuracy of 83% and sensitivity of 88.9%. In a systematic review focused on the prediction of microsatellite instability based on tumor histomorphology using AI, the algorithms for CRC had great performance with the highest standard of 0.972 (21).

## Role of AI in CRC prognostication

### Prediction of patient survival

Prognostic predictors are valuable tools for treatment decision-making by helping clinicians choose the most suitable treatment modality for each patient (74, 75). Survival prediction may be particularly valuable in early-stage CRC, as it can help clinicians decide whether adjuvant chemotherapy is suitable or not. Skrede et al. used CNN algorithms to stratify CRC patients based on survival rate to identify those patients who would likely not benefit from adjuvant chemotherapy versus those patients who would require such treatment (76). Similarly, another CNN algorithm developed by Jiang et al. could predict disease recurrence risk and overall survival for stage III CRC using gradient boosting (74). A CNN algorithm was also used to predict survival based on stromal microenvironment data obtained from HE slides (77). The HE slides were categorized into nine distinct classes, including adipose tissue, background, debris, lymphocytes, mucus, smooth muscle, normal colonic mucosa, cancer-associated stroma, and CRC epithelia. Subsequently, the CNN algorithm was trained, and a deep-stroma score was obtained to determine overall survival as the primary endpoint, and disease-specific survival and relapse-free survival as the secondary outpoints. The resulting model was found to predict these endpoints with a nine-class accuracy of 94%. Our group has also shown that MRI-based texture features of intra-tumor heterogeneity were associated with survival outcomes and improved the performance of standard clinicopathological variables in predicting survival in 55 patients stage IV CRC (78). A random forest ML model found an AUC of 0.83 for the standard clinicopathological prognostic variables and 0.94 when imaging-based heterogeneity features were added.

AI algorithms have not only shown to be capable of predicting survival data, but they may also be able to predict remaining lifespan for advanced-stage CRC. Wang et al. used the Surveillance, Epidemiology, and End Results (SEER) database and tree-based classification to predict whether patients will survive in five years, along with their estimated remaining months if they are predicted to die within five years (75). The model was shown to predict survival with an accuracy of 0.71 and a sensitivity of 0.85. Al-bahrani et al. also used the SEER database and DNN for survival prognostication of CRC (79). The DNN algorithm was trained using patient characteristics including tumor size, age at diagnosis, reason for no surgery, grade, and diagnostic confirmation. The resulting model could predict one, two, and five-year survival with an AUC of 0.87. Finally, Gupta et al. used several ML algorithms including random forests and SVMs to predict tumor stage and disease-free survival based on tumor aggression score (80). An accuracy of 84% was obtained using this model. In one systematic review focused on radiomics for the prediction of treatment outcome and survival, Staal et al. found that the literature had heterogenous methods and included features, but they nonetheless found good performance with respect to predicting response in rectal cancer in robust studies (22).

## Therapeutics: predicting response to therapy

### Personalizing and planning treatment

Planning treatment for colorectal lesions is a multifaceted approach and involves several therapeutic modalities depending on TNM staging criteria, and other clinicopathological characteristics (81, 82). For instance, for patients with stage IV CRC, anti-EGFR, immunotherapy or anti-VEGF may be selected depending on mismatch repair and MSI status (74). In other cases, preoperative neoadjuvant chemoradiotherapy in combination with mesorectal excision might be recommended for T3 and T4 node positive tumors, while T1 and T2 node negative cases may be more suitable for submucosal excision with no preoperative therapy (67, 81). To this end, AI can help in disease staging for treatment planning in patient with CRC. One study conducted by Kim et al. showed that AI could be used to differentiate T2 and T3 rectal cancers (81). Using 290 MRI images from 133 patients, a CNN algorithm was developed to automatically segment and classify tumors as either T2 or T3 with an accuracy of 94%. More recently, Wu et al. utilized faster region-based CNNs to create an automatic diagnosis platform for T staging of rectal cancer *via* MRI (83). The study found AUC of 1 for T1-T4 stages in the horizontal plane and 0.96, 0.97, 0.97, and 0.97 for T1-T4, respectively. In addition to T stage, AI is being increasingly used in lymph node staging for CRC. A recent systematic review and meta-analysis included 17 studies focused on detecting lymph node metastasis in CRC that were published from January 2010 to October 2020 (20). 12 (70.6%) of the studies that met inclusion criteria utilized radiomics models and 5 (29.4%) used deep learning models. The analysis found a per-patient AUC of 0.92 for the deep learning and 0.81 the radiomics models, which were significantly greater than that of the radiologists 0.69 in rectal cancer. Similar results were seen in CRC, where the per-patient AUC in the radiomics model 0.73 was greater than that of the radiologist 0.68. Furthermore, lymph node metastasis is an important consideration for additional surgery in T1 CRCs following endoscopic resection. Ichimasa et al. analyzed 690 patients with T1 CRCs that were surgically resected and developed an AI model with 45 clinicopathological factors to predict presence or absence of lymph node metastasis compared to American, European, and Japanese guidelines (84). The study found a significantly lower rate of unnecessary additional surgery attributable to the false positive detection of lymph node metastasis (AI model: 77% versus 85%, 91%, and 91% in the American (NCCN), European (ESMO), and Japanese (JSCCR) guidelines, respectively; all P < 0.001).

Apart from staging, AI can also aid in the treatment decision-making for CRC *via* personalized evidence-based consulting through supporting systems such as IBM’s Watson for Oncology (WFO). Aikemu et al. evaluated the concordance between treatment recommendations for 250 patients with CRC from WFO and those from a multidisciplinary team at a major center (23). The study found an overall concordance of 91%, and in subgroup analyses, they found overall concordance rates of 83, 94, and 88% for stages II, III, and IV, respectively, and 97, 93, 89, 87, and 100% for neoadjuvant, surgery, adjuvant, first line, and second line treatments, respectively.

### Primary colorectal cancer

A recent systematic review analyzed the use of radiomics in predicting response to treatment for both primary and metastatic CRC (24). The review included 27 studies in primary CRC 2015-2019. All of the included studies focused on the response to chemoradiotherapy. 21 (77.8%) studies obtained radiomic features from MRI. 26 (96.3%) of these studies evaluated the pathologic response to treatment with various methods. While 10 studies evaluated the predictive power of individual radiomic features, most studies either found multiple radiomic feature that were significantly associated with the response to treatment or had developed combination models that were predictive of the response to chemoradiotherapy. Interestingly, four of the identified studies were able to incorporate clinicopathological and/or treatment characteristics into the pre-treatment radiomic features to generate prediction models. For instance, Bibault et al. combined clinical and radiomics features from pretreatment CT scans to create a DNN to predict the complete response to neoadjuvant chemoradiation in 95 patients with T2-4 N0-1 rectal adenocarcinoma (85). The study found that the DNN had an 80% accuracy in predicting complete response and was better than a linear regression model (69.5% accuracy) that used only TNM stage as a predictor and a SVM model (71.58% accuracy) utilizing the same features of the DNN. Cusumano et al. obtained morphological (tumor geometry and shape), statistical (entropy, skewness, and kurtosis), and fractal (tumor heterogeneity) features from the gross tumor volume of T2-weighted pre-treatment MR scans in 198 patients with locally advanced rectal cancer (LARC) (86). They found that most predictive model had accounted for clinical T and N stage. Yi et al. developed a radiomics SVM-based model that incorporated both MRI-based texture analysis and clinicopathological features to predict response to neoadjuvant chemoradiotherapy in 134 patients with LARC (87). The study found that their predictions of pathologic complete response, good-response, and down-staging had high classification efficiencies with AUC of 0.91, 0.90, and 0.93, respectively. Liu et al. also developed a radiomics model that incorporated radiomics signatures and clinicopathologic risk factors, which showed AUC of 0.98 (88).

More recent efforts have focused on predicting pathologic complete response to neoadjuvant chemoradiotherapy in LARC in innovative ways with larger cohorts. Feng et al. utilized a previously validated radiopathomics model, RAdioPathomics Integrated prediction System (RAPIDS), that integrated radiomics features from MR scans and pathomics features from H&E-stained biopsy slides to predict the pathological complete response in patients with LARC (89). They found an AUC of 0.87 in their training cohort of 303 patients, an AUC of 0.86 in their validation cohort of 280 patients, an AUC of 0.81 in another validation cohort of 150 patients, and an AUC of 0.81 in a prospective cohort of 100 patients. Lou et al. developed an AI model by utilizing digital pathological images on 842 patients with LARC and found an AUC of 0.71 in the testing cohort and 0.72 in the external validation cohort (90).

### Metastatic colorectal cancer

A systematic review included six studies in patients with metastatic colorectal cancer, five of which were focused on colorectal liver metastases (24). Most of these studies evaluated response to chemotherapy and radiomic features from CT imaging. However, only three (60%) of the studies found moderate predictive power (AUC 0.74-0.81). Ahn et al. utilized baseline CT texture analysis in 235 patients with colorectal liver metastasis who underwent chemotherapy using FOLFOX and FOLFIRI (91). They found that the lower skewness in 2D showed an AUC of 0.80 and a narrower SD on 3D showed an AUC of 0.79. In contrast, Zhang et al. used baseline MR texture analysis in 26 patients with 193 colorectal liver metastasis and found that a higher variance, entropy, contrast, and a lower angular second moment, correlation, and inverse difference moment were associated with response to chemotherapy with AUCs of 0.60-0.78 (92). In their multivariable logistic regression analysis, variance and angular second momentum could predict lesions that responded to therapy from those that did not. Helden et al. evaluated radiomic features in patients with metastatic CRC who had pre-treatment ^18^F-FDG PET/CT scans and underwent first- or third-line palliative systemic treatment, and they found significant correlations with clinical outcome and select radiomic features (93).

In a more recent systematic review and meta-analysis, Russo et al. analyzed the use of AI in predictive models of the response to cytotoxic chemotherapy alone or combined with targeted therapy in patients with metastatic CRC in 26 original articles (25). In their meta-analysis, which included ten articles, they found that the overall weighted means of the AUC were 0.90 in the training sets and 0.83 in the validation sets. Additionally, the delta radiomics and gene signatures were able to accurately identify up to 99% of patients with metastatic CRC that were responders and up to 100% of patients who were non-responders.

## AI in colorectal cancer surgery

Artificial intelligence has also begun to make an impact in CRC surgery (94). One application of AI is in phase recognition, which involves classifying segments of an operation into predetermined surgical phases. Kitaguchi et al. used CNN-based deep learning for automatic surgical phase recognition on 71 laparoscopy sigmoidectomy cases (95). The study found good accuracy of for the automatic surgical phase recognition (91.9%) and 89.4% and 82.5% for the automatic surgical action recognition of extracorporeal action (89.4%) and irrigation (82.5%). In another study, Kitaguchi et al. developed an annotated video dataset of 50 transanal total mesorectal excision procedure, and their deep learning-based model in automatic surgical step recognition resulted in an overall accuracy for all classification steps of 93.2% (96). Moreover, AI has increasing potential for intraoperative guidance with image-based recognition. Kolbinger et al. recently trained CNNs to discriminate surgical phases, anatomical structures, and tissue planes in 57 robot-assisted rectal resection cases (97). Igaki et al. similarly developed a deep learning-based model that could detect the areolar tissue area in a total mesorectal excision plane with a dice coefficient of 0.84 (98). AI has also allowed for real-time microcirculation analysis of colonic perfusion status *via* indocyanine green angiography to predict anastomotic complications following laparoscopic colorectal surgery (99). Park et al. analyzed and found significantly greater accuracy and consistency in their AI model, which predicted risk of anastomotic complications in patients who underwent laparoscopic surgery for their CRC and that were based on a self-organizing map network, than in the conventional quantitative parameter-based method (99). Mazaki et al. utilized auto-AI model to develop a model that predicted anastomotic leakage in patients who underwent curative surgery for CRC, and they found an AUC of 0.77 (100).

AI has allowed for improvements in surgical training in colorectal surgery. For instance, Kitaguchi et al. generated a 3-D CNN to automate surgical skill assessment in 1480 videos from 74 laparoscopic colorectal surgeries (101). The study found that model was able to automatically classify video clips into screening categories with a mean accuracy of 75.0% and a standard deviation of 6.3%.

Finally, AI has been used to predict outcomes and surgical management of patients with CRC. A recent study by Masum et al. analyzed 4336 patients who underwent colorectal surgery between 2003 and 2019 and built a prediction model for length of stay, readmission, and mortality (102). They achieved an accuracy of 83% with support vector regression algorithms to predict length of stay, an accuracy of 87.5% with a Bidirectional Long Short-Term Memory (BI-LSTM) model that predicted readmission, and an accuracy of 80-96% in their classification predictive modeling predicted three different CRC mortality measures–overall, 31-, and 91-days mortality. A meta-analysis focused on using deep learning networks to analyze videos of laparoscopic procedures found 32 studies with various applications in instrument recognition and detection, phase recognition, anatomy recognition and detection, action recognition, surgery time prediction, and gauze recognition (26).

## Clinical validation, applications, limitations, and future directions

Several ML models have been clinically validated to a limited extent. One machine learning-based model successfully predicted the ability to distinguish patients with metastatic colorectal cancer who showed increased overall survival and time-to-next treatment benefit with FOLFOX chemotherapy from those with decreased benefit in clinical trial cohorts (103). A weakly supervised DL framework that incorporated three separate CNNs was developed to predict the status of molecular pathways and mutations, such as MSI, in CRC from histology images (104). The algorithm was externally validated on the Pathology Artificial Intelligence Platform challenge cohort, which included 47 slides from three centers in South Korea (105). Furthermore, several ongoing clinical trials are currently validating the use of PolyDeep, which is a CADe and CADx model. PolyDeep Advance 1 (106) aims to explore whether PolyDeep is more sensitive than blinded endoscopists to detect colorectal polyps, PolyDeep Advance 2 (107) aims to evaluate whether Polydeep assisted colonoscopy can reduce the rate of missed adenomas in the first withdrawal, while PolyDeep Advance 3 (108) aims to see if Polydeep can improve the adenoma detection rate. In another clinical trial, a gradient-boosted machine learning model was developed and validated on participants in the prostate, lung, colorectal and ovarian cancer screening (PLCO) Trial who were diagnosed with CRC during follow-up to predict the risk of death within 10 years from diagnosis (109). Moreover, one DNN model developed from 326 histopathological slides for automated classification of colorectal polypos achieved an internal accuracy of 93.5% and an accuracy of 87% following external validation in 24 US-based institutions (110). Similarly, another AI-based radiopathomics model was developed to predict pathological complete response to neoadjuvant chemoradiation in LARC, and was verified in two external, retrospective cohorts and in a multicenter, prospective observational study (89).

Although recent strides have been made to validate current AI models, the application of AI systems into clinical practice still requires further investigation (111). It is possible that health information technologies such as AI may be an over-confident approach due to its potential risks and limitations. In particular, there may be inherent biases in the data that is used to train the AI models (111). For instance, racial biases and healthcare inequalities may go unnoticed by AI systems, which may be due to the underrepresentation of minority groups and lack of diversity in the training data. In particular, one AI model for MSI detection had lower performance in a cohort involving a high number of Ashkenazi Jews due to their elevated proportion of the BRAF mutation (69). To this end, there may also be overrepresentation of certain groups and the presence of inter-rater variability in the data-labelling process (111, 112). For this reason, algorithms must be externally validated in which outcomes are reproduced in different contexts to ensure the rigor of the models. Moreover, AI algorithms may not be able to adapt to the evolving nature of real-world data (111). Discrepancy between the training data and subsequent data can create data drifts which may hinder the clinical utility of the algorithm. Other concerns involve ethical responsibility and accountability in the presence of AI errors. There is also a lack of structure and standardization regarding the storage and collection of data (111, 112).

While promising, the use of AI in clinical medicine is still at an early stage (113). One of the biggest limitations with AI is that models tend to be limited by the amount and quality of available labeled data for model development and validation. The generalizability of the models is also based on the type of data used in training. Large datasets that are ethnographically diverse will be required to ensure that models can be applied for decision-making in diverse patient populations. Furthermore, there is a need to establish ethical guidelines before models can ever be widely employed to ensure their appropriate use and access (27). Another issue with the clinical application of AI and ML is the “black box” problem, in which we are able to see the inputs and outputs of a model, but not the variables that are used by the model to generate those outputs. More efforts are needed to make the algorithms, especially deep learning algorithms, interpretable to clinicians and to allow streamlining of data preprocessing (114). Additionally, most of the studies have a retrospective design, and more evidence on the effectiveness of AI is needed from multicenter, prospective studies. Finally, standards need to be established for the required accuracy rates to ensure the safe use and legality of AI technology. Efforts are needed to ensure that sensitive data is kept confidential (115). Nonetheless, the potential of AI in medicine, and specifically CRC is promising. AI is likely to prove beneficial and be closely intertwined with the practice of medicine.

## Conclusion

In conclusion, the use of AI for CRC is highly promising despite being in its early stages of development. AI has exceeding potential to revolutionize the scope of CRC management with substantial progress already made in diagnostics, prognostication, and therapeutics during the past decade. While there remains certain challenges to overcome with regards to the generalizability, validation, and clinical application of these technologies, future developments may eventually lead to improved outcomes and to a paradigm shift in how we care for patients with suspected or diagnosed CRC.

## Author contributions

Conceptualization: AM, ZS, TE, DD, investigation: AM, ZS, TE, writing-original draft preparation: AM, ZS, TE, writing-review and editing: AM, ZS, TE, DD, supervision and project administration: DD. All authors contributed to the article and approved the submitted version.
